# Complications in colorectal surgery: risk factors and preventive strategies

**DOI:** 10.1186/1754-9493-4-5

**Published:** 2010-03-25

**Authors:** Philipp Kirchhoff, Pierre-Alain Clavien, Dieter Hahnloser

**Affiliations:** 1 Department of Visceral and Transplantation Surgery, University Hospital of Zürich, Switzerland

## Abstract

**Backround:**

Open or laparoscopic colorectal surgery comprises of many different types of procedures for various diseases. Depending upon the operation and modifiable and non-modifiable risk factors the intra- and postoperative morbidity and mortality rate vary. In general, surgical complications can be divided into intraoperative and postoperative complications and usually occur while the patient is still in the hospital.

**Methods:**

A literature search (1980-2009) was carried out, using MEDLINE, PubMed and the Cochrane library.

**Results:**

This review provides an overview how to identify and minimize intra- and postoperative complications. The improvement of different treatment strategies and technical inventions in the recent decade has been enormous. This is mainly attributable to the increase in the laparoscopic approach, which is now well accepted for many procedures. Training of the surgeon, hospital volume and learning curves are becoming increasingly more important to maximize patient safety, surgeon expertise and cost effectiveness. In addition, standardization of perioperative care is essential to minimize postoperative complications.

**Conclusion:**

This review summarizes the main perioperative complications of colorectal surgery and influencable and non-influencable risk factors which are important to the general surgeon and the relevant specialist as well. In order to minimize or even avoid complications it is crucial to know these risk factors and strategies to prevent, treat or reduce intra- and postoperative complications.

## Introduction

Colorectal surgery is performed for many diseases such as colorectal cancer, ulcerative colitis, Crohn's disease, mechanical bowel obstruction and recurrent diverticulitis, often resulting in major reconstruction of the gastrointestinal tract. Injury, ischemia, rectal prolapse and proctological disorders may also require large or small bowel resection. Potential risks of colorectal surgery are mainly those of any major abdominal surgery, and usually occur while the patient is still in the hospital. Because of the many indications for and the various extents of colorectal or small bowel resections the rate and spectrum of complications differ.

The lack of consensus on how to define and grade postoperative complications has greatly hampered the evaluation of surgical procedures. A new classification of complications, initiated in 1992 by Clavien and Dindo is based on the type of therapy needed to correct the complication. The principle of the classification is simple, reproducible, flexible, and applicable. The Clavien-Dindo Classification appears reliable and may represent a compelling tool for quality assessment in surgery [1,2].

In general, complications can be divided into intraoperative and postoperative complications. Occurrence of intraoperative complications such as bleeding, bowel injury, ureteral lesions and bladder injuries are caused by intraabdominal adhesions, anatomic problems, the experience of the surgeon and many other factors. Major postoperative complications include wound infection, anastomotic leakage, ileus and bleeding [3].

Only a few recent publications elucidate risk factors for intra- and postoperative complications in colorectal surgery [4-6]. The importance of some risk factors such as age, nutrition status of the patient and experience of the surgeon are becoming more accepted [5,7-9]. In addition, there are many other factors that influence outcome of colorectal surgery which could be modified preoperatively to prevent intra- and postoperative complications.

The aims of this review are to provide an overview of the current literature on complications of colorectal surgery and to describe risk factors and strategies to prevent, treat or reduce complications.

## Methods

A literature search was carried out, using MEDLINE, PubMed and the Cochrane library from 1980 to 2009 using the following terms: complications, risk factors, colorectal surgery, colorectal resection, laparoscopy, surgical site infection, anastomotic leakage, and bowel cleansing. This review is a general overview that provides an update on these topics for the reader.

## Preoperative risk factors

**Figure 1:**
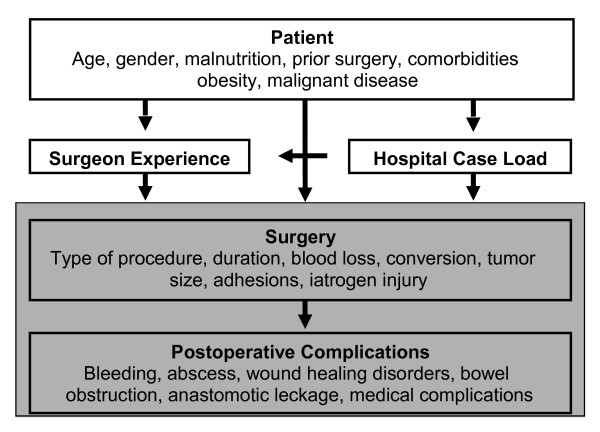
**Risk factors and complications in colorectal surgery**.

Risk factors in emergency, in elective open and laparoscopic colorectal surgery should be recognized prior to surgery in order to reduce complications and to initialize individualized treatment as soon as possible. However, some risk factors such as age, gender and prior abdominal surgery can obviously not be influenced before surgery (Figure 1).

### Non-Influencable Risk factors

#### Age and Gender

In general the postoperative mortality rate in geriatric surgical patients (over 70 years) is low. Despite the increased prevalence of preoperative chronic medical conditions, most patients do well postoperatively. However, the ASA classification (III + IV), emergency surgery, a history of hypertension, pulmonary, neurologic and coronary artery diseases increases the odds of developing any postoperative adverse events in elderly patients [10]. In addition, metastatic disease does increase the postoperative complication rate in patients older than 80 years [11]. Elderly patients who undergo laparoscopic colorectal surgery have a significant shorter length of hospital stay and fewer complications compared to open surgery. Therefore, laparoscopy can be considered a surgical option in all patients regardless of age [12].

Some recent studies showed that male patients have a higher risk of complications in open and laparoscopic colorectal surgery [5]. Male gender is associated with increased anastomotic leakage rates after low rectal anastomoses (see also section below) [13].

#### Prior Abdominal Surgery and Adhesion Formation

In a study of 1000 consecutive laparoscopic colorectal resections patients with prior abdominal surgery had a significantly higher rate of conversion, inadvertent enterotomy, postoperative ileus, reoperation and longer operating times. However, the incidence of other complications and the overall mortality were similar regardless to prior surgical status [14]. After open lower abdominal surgery adhesion related problems and readmission rates were mostly influenced by the initial site of surgery; colon and rectal resections having the highest relative risk of problems directly related to adhesion [15]. The laparoscopic approach seems to decrease postoperative adhesion formation, however long-term clinical studies are lacking [16].

#### Comorbidities

Other predictors of complications are emergency surgery, body weight loss >10% and neurologic comorbidity. A hematocrit <30%, the use of steroids, albumin <3.5 g/L and creatinine >1.4 mmol/L were associated with increased postoperative morbidity and mortality and need to be identified before surgery [17]. In a study of 5'853 patients the following parameters were strongly predictive of perioperative death (overall 5.7%): patients undergoing colectomy because of cancer, ascites, hypernatremia, do not resuscitate status before surgery, ASA classes III-V and a medical history of congestive heart failure. One or more complications were observed in 1,639 of 5,853 (28%) patients. Prolonged ileus (7.5%), pneumonia (6.2%), failure to wean from the ventilator (5.7%), and urinary tract infection (5%) were the most frequent complications. The 30-day mortality rates exceeded 50 percent if postoperative coma, cardiac arrest, a pre-existing vascular graft prosthesis failing after colectomy, renal failure, pulmonary embolism, or progressive renal insufficiency occurred [6].

#### Value of Existing Predictive Risk Scores and Surgeon's Intuition

The risk related to surgery is a function of many factors. Scoring systems to predict morbidity and mortality of various surgeries are important tools for the surgeon and for the patient. These systems generally use data acquired during pre-hospital and in-hospital care, and some supplement this with components measuring the operative severity. Thus, a primary aim of a scoring system is the evaluation of the therapeutic benefit. The existing scoring systems for postoperative morbidity and mortality are the American Society of Anaesthesiologists (ASA), the APACHE scoring system (Acute Physiology and chronic Health Evaluation), POSSUM (Physiological and operative severity score for enumeration of mortality and morbidity), AFC (4-item predictive score of mortality after colorectal surgery) and the Cleveland Clinic Foundation colorectal cancer model. POSSUM calculates expected death and expected morbidity based on 12 physiologic variables and six operative variables. Disadvantages include not taking into account differences among the surgeons, anaesthetists, and operating time; all of which may influence outcome [18-21].

Some studies have tried to predict risk in a less specific manner, and have suggested that a surgeon's gut feeling upon completion of a major procedure may be a good indicator of subsequent outcome. In a study by Hartley et al., an outcome expectation score of 1-3 was a good indicator of the post-operative course of the patient [22]. Another study showed that the surgeon's 'gut feeling' was a good predictor of postoperative outcome in elective surgery but underestimated the risk of complications in the emergency setting. In addition, this study compared the POSSOM score with the surgeon's gut feeling and showed that the POSSOM score overpredicts mortality and morbidity [23].

### Influenceable risk factors

#### Obesity

Initially, it was thought that obese patients have a higher complication rate especially in the case of a laparoscopic approach. However, a few well designed studies have demonstrated that laparoscopic colorectal surgery in obese patients is feasible and safe, and that all known benefits of a minimally invasive approach were preserved [24]. Nevertheless, some groups reported longer operating time, prolonged hospital stay and higher intraoperative complication rates with a higher conversion rate [25]. Although obesity was associated with a high conversion rate, outcome in these converted patients seems to be comparable to open surgery [26]. Patients with a BMI over 25 kg/m^2 ^have a higher risk for incisional hernias and have an increased rate of surgical site infection [27,28]. In elective colorectal surgery, preoperative weight loss is recommended in overweight patients in order to decrease the co-morbidities which are the main cause of complications [5].

#### Nutritional Status

With the availability of improved nutritional supplements and reliable data from well designed meta-analysis on malnourished patients this topic has become more important for every surgeon. Malnutrition has been recognized as an independent risk factor of perioperative morbidity for many decades, but there is currently no standardized definition of malnutrition [29-31]. Depending upon the criteria used for defining malnutrition, its prevalence in gastrointestinal (GI) surgery patients ranges from 30% to 50% [32]. Some scores consist of a questionnaire and others include also blood values (e.g. albumine) [33,34].

A simple score to assess nutritional status based on age, recent weight loss, BMI, severity of disease and planed surgical intervention is the Nutrition Risk Screening 2002 (NRS) or Kondrup Score. A score ≥ 3 is considered as an independent risk factor for complications and perioperative nutritional support should be considered [35].

Various well designed studies have shown beneficial effects of immunonutrition in reducing infectious complications, length of hospital stay, and mortality [36]. It is imperative that the data be interpreted in the context of individual patients risk since specialty formulas appear most beneficial in patients at risk of subsequent complications or those with significant pre-existing malnutrition. Preoperative immunonutrition in malnourished patients was more beneficial than perioperative conventional nutrition support. A total of 305 patients with preoperative weight loss <10% and cancer of the GI tract were randomized into three groups to receive the following: (1) oral supplementation for 5 days before surgery with 1 L/day of a formula enriched with arginine, omega-3 fatty acids, and RNA, with no nutritional support given after surgery (preoperative group, n = 102); (2) the same preoperative treatment plus postoperative jejunal infusion with the same enriched formula (perioperative group, n = 101) (3) no artificial nutrition before or after surgery (conventional group; n = 102). Intention-to-treat analysis showed a 13.7% incidence of postoperative infections in the preoperative group, 15.8% in the perioperative group, and 30.4% in the conventional group [37].

In another study 1,410 GI cancer patients received various types of nutritional support: standard intravenous fluids (SIF; n = 149), total parenteral nutrition (TPN; n = 368), enteral nutrition (EN; n = 393), and immune-enhancing enteral nutrition (IEEN; n = 500). It was noted that nutritional support, particularly IEEN, significantly reduced postoperative morbidity [38].

The postoperative recovery of all surgical patients can be improved by an early start of enteric nutrition postoperatively. When the enteric administration of food is not possible, total parenteral nutrition can be given to bridge a long period without food [39].

#### Preoperative bowel cleaning or not?

Over the last decades the presence of bowel content during surgery has been linked to anastomotic leakage and wound infection. This dogma was based more on observational data than on solid evidence. Several well designed prospective randomized trials have shown that preoperative bowel cleaning does not prevent anastomotic leakage or wound infection in patients undergoing open or laparoscopic colorectal surgery [40-42]. Moreover, one study revealed even an increased risk of anastomotic leaks and wound infection after mechanical bowel preparation. In addition, inadequate mechanical bowel preparation leads to liquid bowel contents and increases the rate of intraoperative spillage [43,44]. Spillage of bowel contents may increase the rate of postoperative infectious complications. On the other hand, bowel preparation might decrease operating time by improving bowel handling during anastomosis and might be helpful when intestinal palpation is necessary for identification of a lesion [45]. In conclusion, bowel preparation is not routinely recommended but should be considered in individual cases, such as when a temporary loop ileostomy is planed.

#### Experience of the Surgeon and Influence of Case Load

Experience is dependent on training, repetitions (learning curve) and on case load (of the surgeon and of the hospital). The learning curve demonstrates the progress in mastering a new surgical technique and is completed when the monitored parameters reach a steady state. In most studies these monitored parameters are operating time, intra- and postoperative complications, conversion rate for a laparoscopic approach, days to discharge, overall morbidity and mortality. The cut off to when a steady state is reached is dependent on many factors and varies greatly with each surgeon. For example, for laparoscopic colorectal resections the learning curve reached a steady state after 30 operations [46]. Another study described 35 cases needed for laparoscopic resection for rectal cancer [47].

When data concerning learning curves are analysed it seems to be important which parameters are monitored. Complication rates, readmission rates and length of stay are more important than the frequently used conversion rates and operating time because the failure of operating time to decline with experience often reflects the surgeons' willingness to attempt more difficult cases rather than an accurate representation of a "learning curve".

The controversy, whether colorectal surgery should be performed by general surgeons or the specialist colorectal surgeons, is gaining increasing importance in Europe. The short and long term results in colorectal surgery as well as in other subspecialties are largely determined by the annual case load and the surgical training in colorectal surgery. Past studies have identified surgeon- and institution-related characteristics as prognostic factors in colorectal cancer surgery.

The COLOR trial (n = 536) investigated the impact of hospital case volume on short-term outcome after laparoscopic surgery for colon cancer. In centers with low (< 5 procedures/year), medium (5-10/year), and high (> 10/year) case volumes, median operating time (240, 210 and 188 minutes; p < 0.001) and conversion rates (24% vs 24% vs 9%; p < 0.001) decreased significantly. Postoperatively, fewer complications (p = 0.006) and a shorter hospital stay (p < 0.001) were observed in patients treated at hospitals with high caseloads. Mortality, however did not differ [48,49].

The Norwegian rectal cancer trial investigated long-term outcome and found that the rate of local recurrence was significantly higher and the survival significantly lower for hospitals with a low annual caseload (<10 procedures per year) than for hospitals with a volume of 30 or more cases [50]. In a Swiss study of 915 patients with 376 rectal and 539 colonic primaries both the surgeon's and hospital's annual caseloads were independent, beneficial prognostic factors for overall survival (P = .0003, P = .044), disease free survival (P = .0008, P = .020), and marginally significant factors for local recurrence (P = .057, P = .055) [51].

Due to increased quality assessment in hospitals and elevated demand for minimal complication rates in surgery the centralization of special colorectal procedures will be most likely implemented in most of the world in the near future.

#### Preoperative Anemia

There is a high incidence of preoperative and postoperative anemia in patients undergoing major non cardiac surgery, with a coincident increase in blood utilization. Studies show that perioperative anemia is associated with increased postoperative complications and mortality [52]. In a recent study the 30-day mortality and cardiac event rates increased, with either positive or negative deviations from normal hematocrit levels [53]. Consideration should be given to preoperative diagnosis and correction of anemia with iron, vitamin B12, and folate supplementation. The results of erythropoietin administration remain controversial [54].

## Intraoperative risk factors

### Open access to the abdominal cavity

The choice of incision for laparotomy depends on the area that needs to be exposed, the elective or emergency nature of the operation and the personal preference. The transverse incision seems to be associated with fewer early postoperative complications (mainly pain and pulmonary morbidity) and lower incidence of late incisional hernia [55]. However, some authors report abdominal or neural dysfunction after transverse access because of nerve, muscle or vessel interruption [56]. A midline incision is still the incision of choice in conditions that require rapid intra-abdominal entry or where the preoperative diagnosis is uncertain, as it is quicker and can easily be extended [57].

### Laparoscopic Access to the Abdominal Cavity

Abdominal access in endoscopic surgery carries a finite risk of visceral injury. Bleeding, intestinal perforation, vascular injury, intraperitoneal adhesions and subcutaneous emphysema are the main potential complications. To date there are four techniques used to create a pneumoperitoneum: blind Veress needle, direct trocar insertion, optical trocar insertion and open laparoscopy. The first two entry mechanisms are blind. The described overall complication rates in these techniques are below 1%. Some studies revealed a higher rate of visceral injuries in the open-entry technique. In a survey 106 gynecologists (57%) used only the closed-entry technique. This group reported 31 complications (0.1%) in 31,532 procedures. Even in the case of patients who were at risk for entry-related complications (previous laparotomy, obesity), pneumoperitoneum was established by the closed-entry technique. The remaining 81 gynecologists used both entry techniques. However, the open-entry technique was used on special indications and in only 2.0% of cases (range: 1-20%). These special indications were suspected adhesions or previous laparotomy (90%) and obese (7%) or very thin patients (3%). These 81 gynecologists reported 20,027 closed-entry procedures and 579 open-entry procedures and complication rates of 0.12% and 1.38%, respectively (P < 001). Significantly more visceral lesions were found (P < .001) at open-entry technique [58]. Therefore there is no evidence to prefer one technique in laparoscopic access.

To select the kind of access a recent study gives some useful recommendations: (1) use left upper quadrant entry in patients with suspected adhesions or umbilical hernia (2) limited movement of the inserted Veress needle (3) an intraperitoneal pressure less than 10 mmHg is a reliable indicator of correct placement of the Veress needle (4) the angle of the Veress needle should be at entry 45 degrees in non-obese patients and 90 degrees in obese patients. Direct insertion of the trocar without prior pneumoperitoneum is associated with less insufflation-related complications such as gas embolism, is faster to perform and is a safe alternative. The visual entry cannula system may provide advantages over the traditional techniques but has to be fully explored in the future [59].

The seriousness of vascular injury is high in comparison to visceral injuries during the abdominal access. These cases are rare and no evidence based recommendations of treatment can be given. Injuries to the main vascular structures need an immediate conversion and surgical repair. Small bowel injuries can be treated laparoscopically. Severe lesions sometimes require segment resections and conversion to open surgery. Injuries of the liver or spleen are manageable with laparoscopic devices. If severe bleeding continues a pre-emptive laparotomy is recommended (see also next section).

### Iatrogenic Injuries and How to Handle Them

There is limited data regarding iatrogenic injuries in colorectal surgery. The main fears of the surgeon are vessel injury, damage to the spleen during colorectal surgery (incidence of 0.006%) [60], or intestinal perforation and uretric injuries (incidence < 0.01%). Injuries to the abdominal or pelvic veins occur mainly in patients undergoing oncologic resections, and those with difficult anatomic exposure, owing to previous operation, recurrent tumor or radiation therapy. Most of the injuries can be repaired by primary suture or end-to-end anastomosis. Few injuries need interposition grafts, patch venoplasty or venous ligation. Therefore a vascular surgeon should be available in hospitals where cancer resections are frequently performed [61].

Iatrogenic perforation of the bowel occurs either during adhesiolysis or inadvertently due to thermic lesion, the latter are often not recognized during the operation. The surgeon should prefer primary repair or resection with anastomosis. In laparoscopic cases the bowel injury should be sutured immediately as it might be difficult to localise later [62,63].

In general, the incidence of iatrogenic splenic injury is underestimated because of poor documentation. Splenectomy is considered a poor prognostic factor [55,64]. Splenic injury results in increased blood loss, longer hospital stay and higher mortality and infection rates. Splenic injury can be reduced by achieving good exposure, avoiding undue traction and careful division of splenic ligaments and adhesions. If the spleen is injured preservation is desirable and often feasible [65].

### Which Instruments Help the Surgeon and Which May Harm the Patient?

To date, data available concerning studies which compare the safety of surgical devices are limited. Conventional monopolar electro-surgery has several short-comings in laparoscopic surgery including the risk of thermal injury, difficult hemostasis and disturbing smoke production, making the use of additional tools like bipolar graspers, sutures or clips necessary. To overcome these problems and to reduce instrument changes, number of trocars and operation time, several multifunctional tools have been developed. The most popular devices are electro thermal bipolar vessel sealers and ultrasonically coagulating shears. In a recent prospective randomized study we could show that bipolar vessel sealers and ultrasonic coagulation shears shorten dissection time in laparoscopic left-sided colectomy and are cost-effective compared to monopolar electro surgery. Other studies showed less operative blood loss and a decrease in operating time when the ultrasonic dissection device were used. For now it is still the preference of the surgeon as to which device is used [66,67].

### Intraoperative Blood Loss and its Influence on Postoperative Outcome

Preoperative anemia and intraoperative blood transfusion are independent risk factors for intra- and postoperative complications in colorectal surgery [5]. In a case-matched study of 147 patients undergoing colectomy using either an open or laparoscopic approach the open colectomy group required significantly more units of blood (P = .003) to maintain similar hemoglobin levels after surgery. Estimated blood loss (P < .001) and the number of patients who received transfusions on the day of surgery (P = .002), during the first 48 hours after surgery (P = .005), and during the entire hospital stay (P = .003) were significantly higher in the open colectomy group [68].

To prevent intraoperative blood loss and postoperative complications some laparoscopic surgeons prefer ultrasonic dissection with produces significantly less blood loss and thereby iron supplementation in preoperative anemic patients two weeks prior to surgery [69].

### Conversion A decision for the Patient's Safety or a Failure of the Surgeon?

One of the initial arguments to discredit laparoscopy was the index of conversion, which was interpreted as operative failure. However, today conversion is no longer considered a failure, but as result of good clinical judgment. The average conversion rate in laparoscopic colorectal surgery is approximately 10%. Independent predictive risk factors for conversion are BMI (odds ratio of 2.1 per 10 units increase in BMI), ASA grade (I 2.3%, II 9%, II-IV 13.8%), type of resection (low rectal resection 18.4% vs. left colorectal resection 15.3% vs. 8.1% in right colon resections), intraoperative abscess, surgeons seniority [70]. The Laparoscopic Colorectal Surgery Study Group showed in a multicenter study with 1'658 patients a conversion rate of only 5.2% (n = 86). Converted patients were significantly heavier (body mass index 26.5 vs. 24.9) and rectal resections were converted more frequently (20.9 vs. 13 percent) [71].

Specific indications for conversion were technical problems, adhesions, bleeding, abscess, fistula, inflammatory mass and bowel perforation. Also prior abdominal surgery increased the conversion rate up to 41% in a recent study [14,72]. The effect of conversion on morbidity and mortality is discussed controversially in the literature. Recent studies describe similar outcome after conversion compared to the open access [71,73]. However, large randomized trials clearly demonstrate increased morbidity and loss of short-tem benefits in converted patients [49,74]. Obesity is associated with a higher conversion rate but the outcome of converted patients seems to be similar to the open cases [26]. In another study obesitiy was not a risk factor for conversion [75].

### Drainage in elective colorectal anastomoses?

The value of prophylactic drainage in colorectal surgery has been studied extensively. Currently available data from randomized controlled trials point out that a routine prophylactic drainage provides no benefit after uncomplicated major colon and rectal surgery [76]. On the contrary, a no drain policy was associated with less wound infections and a fewer anastomotic leaks. These studies underscore the low sensitivity of drains in detecting leakage and bleeding, which questions the putative warning function of a prophylactic drain. In addition, neither acute/simple nor gangrenous or perforated appendicitis benefit from a prophylactic drainage. In summary, there is sufficient evidence showing that routine drainage after colorectal anastomoses does not prevent leaks or other complications [77,78].

### When Do We Need a Protective Stoma?

A stoma may be a temporary solution when there is a dysfunction of a colostomy or ileostomy however the advantages are still under debate. One group argues that a protective stoma is only indicated in low rectal resections in patients with significant comorbidities, neoadjuvant radiochemotherapy and feculent peritonitis [79]. If adjuvant radiochemotherapy is considered postoperatively in patients with a colorectal carcinoma the closure of the temporary loop ileostomy should be performed before the chemotherapy to minimize complications [80]. Other groups do not recommend a stoma at all or only when the colorectal anastomosis is in the lower third of the rectum [81,82]. In emergency situations with peritonitis and perforation of the left colon primary anastomosis and protective ileostomy should be performed rather than a Hartman's procedure [83,84].

### Operating Time

The duration of the operation is influenced by many factors such as; surgical technique (open or laparoscopic), intraoperative complications, prior abdominal surgery, surgeon's experience and the operating team. Many studies showed that prolonged operating time correlated with higher intra- and postoperative complications. In a series of 541 colorectal anastomoses between 1999 and 2004 at a single colorectal unit, univariate analysis showed that a prolonged operating time had an odds ratio of 2.8 for developing an anastomotic leakage [13].

On the other hand, nearly all prospective randomized studies comparing a laparoscopic with the open approach reported longer operations in the laparoscopic group, but surprisingly without an increase of intra- and/or postoperative complications and with similar morbidity and mortality rates [85,86]. Probably, the negative effect of the prolonged operating time in laparoscopic surgery is overrun by advantages such as decreases in-hospital stay, wound infection, postoperative ileus and postoperative pain. However, there is a lack of well designed studies evaluating the influence of the operating time on postoperative outcome as a primary endpoint.

## Postoperative Factors

The majority of advancements in the care and survival of surgical patients have occurred in the postoperative period. These advances include changes in postoperative feeding, activity, pain control, ulcer and deep venous thrombosis prophylaxis. Here, we give a short update of current trends in postoperative analgesia and diet.

### Postoperative Analgesia

The decreased length of hospital stay due to more cost effective outpatient procedures necessitates good postoperative pain management. It has been demonstrated that well managed pain control supports respiratory function and lowers the risk of complications [87]. In colorectal surgery the major modalities of postoperative pain control are patient-controlled anaesthesia, opioids, nonsteriodal anti-inflammatory drugs, and epidural anaesthesia.

Some studies show that pain control, patient satisfaction and bowel function are improved after abdominal surgery under epidural analgesia [88]. Carli et al. showed in a prospective randomized study that epidural anaesthesia significantly shortened the duration of postoperative ileus and improved postoperative pain control. Postoperative complication rates and length of hospital stay was not shown to be improved in this study [89]. Another study demonstrated that continuous epidural analgesia is superior to patient controlled opioid analgesia in relieving postoperative pain for up to 72 hours, but was associated with a higher incidence of pruritus [90]. Epidural anesthesia has a low complication rate, however, if complications occur they are mostly severe. The risk of a symptomatic spinal mass lesion after patient-controlled epidural analgesia was 1:2857 (0.04%), including epidural haematoma (0.02%; 1:4741) or epidural abscess (0.014%; 1:7142). Another recent study demonstrated that epidural analgesia reduced the need for prolonged ventilation or reintubation, improved lung function, increased blood oxygenation, reduced risk for pneumonia, to the contrary increases the risk of hypotension, urinary retention, and pruritus. Technical failures occurred in 7% [91,92]. Despite the advantages of epidural anesthesia its use alone cannot prevent postoperative morbidity and mortality. It is therefore necessary to address its use in the context of multimodal intervention.

### Postoperative Diet

The resumption of a diet is critical to the recovery. Before discharge patients should demonstrate return of intestinal tract function based on oral food intake, flatus and/or bowel movements. Traditionally, patients received a nasogastric tube decompression and were set on a "nil per os diet" postoperatively. Different trials failed to show that a nasogastric tube has any postoperative benefits for the patient, causing most surgeons to abandon its routine use [93]. There is much variability in regards to restarting enteral nutrition in patients undergoing colorectal surgery. Several trials demonstrated that the majority of patients tolerated oral intake in the immediate postoperative period, regardless of the presence or absence of traditional markers of normal gastrointestinal function. In a metaanalysis of 837 patients it was seen that reduced postoperative infections, reduced anastomotic complications and shorter length of stay was shown in patients who received immediate postoperative normal diet compared to patients who were fasted until gastrointestinal functions were resumed [94]. In a recently published analysis the advantages of early enteral feeding were not significant but showed a trend towards fewer postoperative complications [95]. Another metaanalysis of 13 trials (1'173 patients) came to the conclusion that there is no obvious advantage in keeping patients 'nil per os' following gastrointestinal surgery. Early enteral nutrition was associated with reduced mortality. This review supports the notion that early commencement of enteral feeding may be of benefit compared to the nothing by mouth policy [96].

In the last few years Kehlet et al. favored a multi-modal rehabilitation with an emphasis on preoperative information, reduction of surgical stress responses, optimized dynamic pain relief with continuous epidural analgesia, early mobilization and oral nutrition (fast-track surgery) [97]. Current results from fast-track colonic surgery suggest that postoperative pulmonary, cardiovascular, and muscle function are improved and body composition preserved as well as a normal oral intake of energy and protein can be achieved. Consequently, hospital stay is reduced to about 2-4 days, with decreased fatigue and need for sleep in the convalescence period. Despite a higher risk for readmissions, overall costs and morbidity seem to be reduced [97-99]. A recent randomized study by our group compared the 30-day complication rate of patients who underwent a fast track protocol or standard care after open colonic surgery. The fast-track protocol significantly decreased the number of complications (16 of 76 in the fast-track group vs. 37 of 75 in the standard care group; P = .0014), resulting in shorter hospital stay (median, 5 days; range, 2-30 vs. 9 days, respectively; range, 6-30; P < .0001). Fluid restriction and effective epidural analgesia were the key factors that determine outcome in the fast-track program [100].

In summary, there is a growing body of evidence that early enteral nutrition improves outcome and reduces postoperative complications. Despite proven advantages of fast track surgery the implementation of a standardized and multidisciplinary care is difficult since resistance is still enormous.

## Complications

The most frequent postoperative surgical complications after colorectal resections are surgical site infection, anastomotic leakage, intraabdominal abscess, ileus and bleeding (Figure 1). These complications have different influences on outcome and have to be diagnosed accurately. In order to meet certain quality standards it is essential to assess postoperative complications [101].

### Surgical site infection (SSI)

Colorectal operations are, at best, clean-contaminated procedures, and at times there is contamination of both the peritoneal cavity and the surfaces of the surgical wound. In addition, the diseases of the large bowel that require surgery tend to afflict elderly patients. Collectively, the combination of an unclean environment, major surgery and debilitated patients creates a situation that is associated with a very high incidence of wound infection. In open colorectal surgery the incidence of SSI varies from 2-25% and is associated with BMI ≥ 30, creation/revision/reversal of an ostomy, perioperative transfusion, male gender, ASA Score ≥ III and wound contamination [102,103]. The incisional SSI rates in colon (n = 339) and rectal (n = 217) resections were 9.4% and 18.0%, respectively (P = 0.0033). Risk factors for SSI in colon surgery were ostomy closure (OR = 7.3) and lack of oral antibiotics (OR = 3.3), while in rectal surgery, risk factors were preoperative steroids (OR = 3.7), preoperative radiation (OR = 2.8), and ostomy creation (OR = 4.9) [104]. Some studies showed that perioperative oxygen supply and preoperative immunonutrition decreased SSI significantly [105,106]. It is widely accepted that a laparoscopic approach lowers the rate of SSI [36,107]. As for laparoscopic appendectomies [108], most surgeons use plastic wound protectors during specimen removal after laparoscopic resection. This certainly facilitates extraction through a small incision, but there are no randomized controlled trials demonstrating a reduction in wound infection. The role of antibiotic prophylaxis in preventing postoperative complications in colorectal surgery is well established through many studies. However, there is still a debate about the duration of the antibiotic treatment and the kind of antibiotic which should be used. In summary, most studies favour one to three intravenous doses of a second generation cephalosporine with or without metronidazole with the first dose being administered before skin incision [109,110].

### Anastomotic leakage: Risk factors, diagnosis and treatment

Anastomotic leakage is the most serious complication specific to intestinal surgery and ranges from 2.9% to as high as 15.3%. At least one third of the mortality after colorectal surgery is attributed to leaks. Within this context, knowledge of factors influencing anastomotic healing appear even more important [81,111]. However, there is lack of a clear definition for what constitutes an anastomotic leak (radiological proven, clinically relevant, with or without abscess).

In general, the leakage rate for intraperitoneal anastomoses is significantly lower than for extraperitoneal anastomoses. Anterior rectal resections have the highest leakage rate of up to 24% [112,113]. The main risk factors for anastomotic leakage using univariate analysis were male gender (OR = 3.5), previous abdominal surgery (OR = 2.4), Crohn's disease (OR = 3.3), rectal cancer < or = 12 cm from the anal verge (OR = 5.4) and prolonged operating time (*P *= 0.05 as a continuous variable and P = 0.01 when prolonged operative time was >120 min). Male gender, a history of previous abdominal surgery and the presence of a low cancer remained significant after multivariate analysis [13].

Another multivariate analysis showed that American Society of Anaesthesiologists Grade III to V (P = 0.04; odds ratio, 5.6; 95 percent confidence interval, 1.6-15.3) and emergency operation (P = 0.03; odds ratio, 4.6; 95 percent confidence interval, 1.9-9.8) were independent factors associated with anastomotic leakage. The risk of anastomotic leakage was 8.1% (odds ratio, 10.5; 95% confidence interval, 2.7-26.8) if both factors were present [114].

Most studies comparing high and low anterior resections have shown that the level of anastomosis is the most important predictive factor for leakage. The high-risk level for leakage varies between anastomoses from <10 to <5 cm from the anal verge depending on the cited study [115,116].

There seems to be no significant difference in leakage when comparing a handsewn and a stapled technique regardless of the level of anastomosis [117]. Intraoperative problems and postoperative strictures seem to be more frequent in stapled anastomosis [118]. However, in a recent Cochrane review ileocolic stapler anastomoses were associated with fewer leaks than handsewn anastomoses [119].

The available data comparing the anastomotic leakage rate in laparoscopic or open operated patients showed no difference regardless of the level of the anastomosis [120]. In cancer patients anastomotic leakage (regardless of open or laparoscopic technique) is associated with poor survival and a higher recurrence rate after curative resection [121,122].

### Diagnosis of anastomotic leakage

Because of the severity of the complications associated with an anastomotic leak, it is imperative to identify the problem and act as early as possible. Most groups base the diagnosis on clinical symptomatic leakage, manifested as gas, purulent or fecal discharge from the drain, purulent discharge from the rectum, pelvic abscess or peritonitis. It is usually necessary to obtain objective tests of anastomotic integrity because of the non-specific clinical signs. Water soluble enemas or CT scans are widely used for diagnosis of anastomotic leak. Interestingly, in two recent studies anastomotic leaks were more often diagnosed late in the postoperative period and more often after hospital discharge, or 12 days postoperatively [123,124].

### Treatment of Anastomotic Leakage

Anastomotic leaks may be divided into those which are clinically significant and those which are not. Subclinical leaks are more benign in their natural history compared with clinical leaks although quality of life and bowel function does not differ in these groups [125]. In pelvic abscess formation after colorectal surgery CT scan-guided percutaneous drainage should be performed in hemodynamically stable non-septic patients and has a success rate of up to 80% [126,127]. With signs of free anastomotic leckage in the abdominal cavity by CT scan the indication for surgery is mostly given. Despite the good results with conservative therapy (including antibiotics), the indication for surgical repair of anastomotic leakage should be made as early as possible to improve patient outcome. Re-laparoscopy and lavage after laparoscopic operation is feasible and safe and has less postoperative complications than an open re-intervention [128].

### Postoperative Bleeding

In general postoperative bleeding after colorectal procedures is a rare complication. The risk depends on the performed surgical procedure, the co-morbidities of the patient and in individual cases on an impaired clotting system. In the initially postoperative phase abnormal heart rate and low blood pressure should be reported and interpreted by the surgeon. Haemoglobin and hematocrit measurements can help to determine a blood loss.

### Ileus

Postoperative ileus has long been considered an inevitable consequence of gastrointestinal surgery. It prolongs hospital stay, increases morbidity, and adds to treatment costs. The pathophysiology of postoperative ileus is multifactorial. The operating time and intraoperative blood loss are independent risk factors for a postoperative ileus [129].

Postoperative ileus can develop after all types of surgery including extraperitoneal surgery. A variety of treatment options have been reported. However, it is difficult to compare these studies because of of the different anesthesia protocols used and patient comorbidities differed significantly.

Paralytic postoperative ileus is usually treated with a combination of different approaches. These include limitation of narcotic use by substituting alternative medications such as nonsteroidals and the placement of a thoracic epidural with local anesthetic. The selective use of nasogastric decompression and correction of electrolyte imbalances also are important factors to consider.

## Conclusion

Here, we summarize the main complications of colorectal surgery which are important to the specialist, the general surgeon and the gastroenterologist as well. We also tried to show strategies to minimize intra- and postoperative complications. Development in treatment strategies and technical inventions in the recent decade have been enormous. This is mainly due to the laparoscopic approach, which is now well accepted. Training of the surgeon, hospital volume and learning curves are becoming more important to maximize patient safety, evaluate surgeon expertise and calculate cost effectiveness. In addition, standardization of postoperative care is essential to minimize postoperative complications. Risk factors which influence intra- and postoperative complication rate are summarized in Table 1.

**Table 1:** Importance of risk factors for intra- and postoperative complications

Risk factors	Intraoperative Complications	Postoperative Complications
Age	+	+

Male Gender	+	+

Malnutrition	+	+

Experience Surgeon	(+)	+

ASA class > III	+	+

Preoperative anemia	(+)	+

Intraoperative blood transfusion	+	+

Operating time	+	+

Neoplasia	+	+

Bowel injury	+	+

Obesity	+	(+)

Prior myocardial infarction	(+)	+

Heart failure	(+)	+

## Competing interests

The authors declare that they have no competing interests.

## Authors' contributions

PK has made substantial contributions to conception and design, acquisition of data and interpretation of data. DH has been involved in drafting the manuscript and has given final approval of the version to be published. PAC has revised the manuscript critically for important intellectual content and participated in its design. All authors read and approved the final manuscript.
